# Cryogenic Pretreatment Enhances Drying Rates in Whole Berries

**DOI:** 10.3390/foods13101524

**Published:** 2024-05-14

**Authors:** Esperanza Dalmau, Monica Araya-Farias, Cristina Ratti

**Affiliations:** 1 Department of Chemistry, University of the Balearic Islands, Ctra. Valldemossa, km. 7.5, 07122 Palma de Mallorca, Spain; 2 CEA, INRAE, Medicines and Healthcare Technologies Department (DMTS), Paris-Saclay University, SPI, 91190 Gif-sur-Yvette, France; 3 Département des Sols et de Génie Agroalimentaire (SGA), FSAA, Université Laval, Québec, QC G1V 0A6, Canada; cristina.ratti@fsaa.ulaval.ca

**Keywords:** berries, epicarp, drying pretreatments, cryogenic fluids

## Abstract

The impact of cryogenic pretreatments on drying performance was studied in blueberries, seabuckthorn fruits and green grapes. The fruits were immersed in liquid nitrogen in 2 min freezing/thawing cycles (one to five). Untreated samples were used as the control. Drying experiments were carried out on treated and non-treated berries at 50 °C and 1 m/s (hot-air-drying), 50 °C and 25″ Hg vacuum (vacuum-drying), 30 mTorr total pressure and 25 °C shelf temperature (freeze-drying). The weight loss evolution of the foodstuffs was measured as a function of time. Microscopic (SEM and optical) determinations of the epicarp were performed. A visual inspection was performed and color changes and volume reductions were assessed before and after dehydration. The thickness of the berries’ epicarp decreased between 20 and 50% (depending on the fruit) after 3–5 immersions in liquid N2. The drying kinetics was accelerated significantly for the three tested drying processes (i.e., drying time decreased from 48 to 16 h for blueberry freeze-drying). The best quality of dried berries was observed for pretreated blueberries after freeze-drying, keeping their volume, shape and color after the process. This work shows that “tailor-made” dried berry products with desired properties can be achieved and drying performance can be improved by the application of ultra-low temperature pretreatments.

## 1. Introduction

Small fruits such as berries are nowadays known to have great health benefits from potential anti-aging and anti-inflammatory effects, its consumption delaying or preventing the onset of cancer and neurodegenerative diseases [1,2]. Berries are small edible fruits consumed most commonly as a ‘whole’. Thus, the drying of this type of fruit does not require a mechanical reduction in their size prior to processing as is the case for apples or bananas, and usually, they are dried as they are, which presents a technical problem due to their waxy impermeable cuticle (‘skin’) impeding moisture loss.

**Figure 1:**
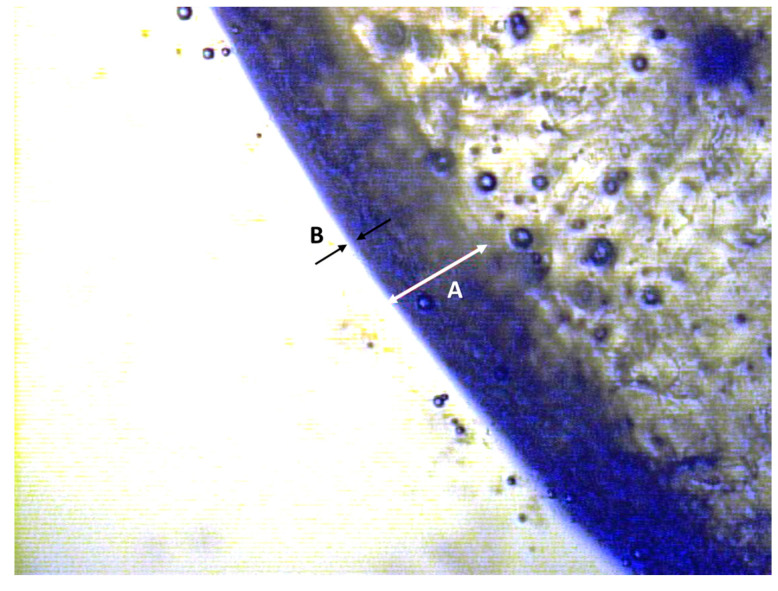
Cross-section of a blueberry fruit (photo is a property of the authors). A: epicarp, B: proper cuticle.

Figure 1 (photo is a property of the authors) shows a cross-sectional diagram of a blueberry displaying the epicarp (commonly known as ‘skin’, marked with A in Figure 1), which consists of a layer of compacted epidermal cells lying below a second layer: the proper cuticle (estimated as B in Figure 1). The cuticle is a noncellular lipoidal membrane forming a major barrier to water and solute movement into and out of plants [3]. The moisture permeability of fruit skin depends on composition, microstructure, the crystalline or amorphous state of the matrix and the lipid and glass transitions occurring during cooling or heating [4]. The cuticle is composed of a biopolymer, the cutin, with embedded intracuticular waxes; further waxes may be deposited on the surface of the cuticle as an epicuticular wax layer [5], and in many fruits, crystalline wax structures are extruded to the external surface, giving fruits their characteristic waxy bloom [3]. The presence of wax in the cuticle is the main reason why whole small fruits, such as grapes, cranberries or blueberries, have an extremely slow drying rate and require skin pretreatments prior to dehydration in order to accelerate moisture loss.

The pretreatment of a material prior to drying has been long used as a technique to accelerate drying rates as well as to improve final product quality. Chemical, thermal and mechanical are traditional pretreatments used to overcome the water barrier present in the waxy skin during the drying of cherry tomatoes, grapes, plums, blueberries and cranberries [6,7,8] and to inactivate enzymes through blanching [9,10]. Although chemical and hot thermal pretreatments are very effective in improving drying rates, these methods have shown a negative impact in the sensory and physicochemical quality of the product [6]. Blanching causes a leak of soluble compounds together with adverse structural changes [9]. In other cases, fruit integrity and leaking are important issues when applying severe mechanical pretreatments [11]. More recent technologies such as high pressure, pulsed high-intensity electric fields and supercritical carbon dioxide have been studied as pretreatments to improve the drying rates of mangoes, bell peppers, etc., with good results [12,13,14,15]. They have been applied to pieces of fruits and vegetables and not to the drying of whole berries.

Freezing is one of the widest used preservation methods due to the extended shelf life of products as well as an improved quality compared to others. The freezing rate has a marked impact on food quality properties, most of the published information indicating that the preservation of quality in cellular food systems is only enhanced by rapid cooling [16,17]. The size and shape of ice crystals are critical for the final quality of frozen foodstuffs, the rate of heat removal being one of the main factors determining the crystal growth rate [18]. Slow freezing helps the formation of large extracellular ice crystals damaging vegetable tissues, while rapid freezing promotes intensive nucleation and the formation of intracellular small ice crystals [19]. Freeze/thaw cycles results in the rupture of water-retaining membranes within fruits tissues [20], eggplant pulp [21] and mango [22] and the freeze injury of the phospholipid bilayers of liposomal membranes [23]. Rapid freezing at cryogenic temperatures results in freeze fractures and cracking in food tissues [24,25,26]. All these reported effects of freezing on vegetable and fruit tissues can certainly be used to induce positive changes in the berry skin microstructure to increase drying rates or improve their final quality.

Most of the traditional research on the impact of freezing as a pretreatment prior to dehydration was conducted for slow freezing [27,28], even if rapid cooling to cryogenic temperatures has proved to be a superior freezing method in terms of quality attributes. More recently, attention has been drawn to the liquid nitrogen spray quick freezing of berries and other edible products [29,30,31], these articles mainly focusing on quality attributes after thawing compared to traditional slow freezing (i.e., better nutrient retention, more uniform water distribution, increased hardness and less damaged cellular microstructure). No studies have been performed on using liquid nitrogen spray quick freezing pretreatment to increase drying rates in berries. Cyclic liquid nitrogen immersions, on the other hand, were used to successfully increase the osmotic dehydration rates of seabuckthorn berries [32] and blueberries [11]. Losses of hydrosoluble compounds (vitamin C, anthocyanins and phenolics) were observed along with the increase in water loss during osmotic dehydration.

The objective of the present research is therefore to investigate the effectiveness of a rapid freezing pretreatment, liquid nitrogen immersion, of whole berries (seabuckthorn, blueberries and green grapes) on the drying performance of hot-air-drying, vacuum-drying and freeze-drying and dried berry quality.

## 2. Materials and Methods

### 2.1. Materials

Blueberries (BB), seabuckthorn fruits (SB) and green grapes (GG) were used for the experiments since they are small fruits with an impermeable epidermis. Blueberries (*Vaccinium corymbosum* L., ‘highbush’, variety) and green grapes were bought from the local market, while seabuckthorn fruits (var. Indian Summer) were harvested in a farm located in Ste-Anne de Beaupré (Québec, QC, Canada). The fruits were cleaned, individually frozen at *−*18 °C, put in 2 kg bags and kept in a cold at *−*40 °C storage (Sanyo MDF-235 chest medical freezer, Gunma, Japan) for at least one week prior to use.

### 2.2. Methodology

**Figure 2:**
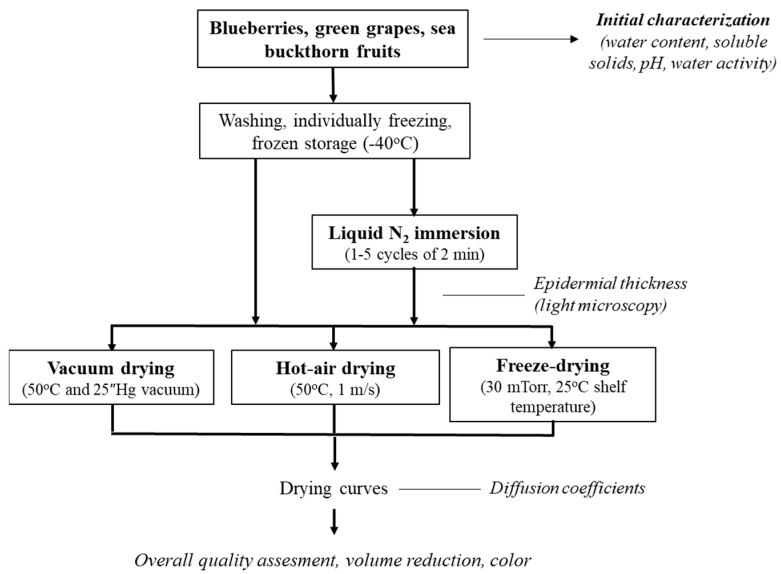
Schematic diagram of experimental protocol.

Figure 2 shows a graphical scheme of the experimental protocol used in this study. Initially, frozen fruits (conditioned as described in the previous section) were pretreated by liquid nitrogen immersion (LNI). Treated and non-treated (control) samples were subsequently subjected to vacuum-drying (VD), hot-air-drying (AD) and freeze-drying (FD).

The impact of LNI on epidermal thickness was assessed through light microscopy, while an overall visual inspection was performed and color changes and volume reductions in the dried samples observed to assess product quality. A description of the particular operations and methods follows in the next few paragraphs.

#### 2.2.1. Liquid Nitrogen Pretreatment

Frozen fruits were immersed in liquid nitrogen in cycles of 2 min inside the cryogenic fluid (−196 °C), followed by 2 min in ambient air (20 ± 2 °C). The cycle was repeated 1 to 5 times. Fruits without immersion in liquid nitrogen were used as the control samples.

#### 2.2.2. Light and Scanning Electron Microscopy

As described previously, the skin (epicarp, A in Figure 1) of a fruit consists of a layer of cells (epidermis) covered by a thin film of cutin (proper cuticle, estimated as B in Figure 1). To assess the epicarp thickness, light microscopy of the berries was performed prior and after LNI pretreatment and thickness changes were estimated from the images.

For the light microscopy tests, a skin sample was prepared from fresh grapes, blueberries and seabuckthorn fruits by cutting a cube with sides of 3 mm from the equatorial area of the berry with a sharp razor blade and then placed flat on microscopy slides. An optical microscope (LEITZ Laborlux S, Wetzlar, Germany) was used to assess the berry skin thickness (epicarp) before and after the LNI pretreatment. The microscope was optimized with a magnification of 40×, light regulated at 5 V, and the option mapping HE-1 to nuance colors. Images were captured using the digital camera provided with the microscope. Epicarp thickness was estimated from images by using the ImageJ 1.53 k software program [33]. The estimations were carried in triplicate.

The surfaces of the berries were analyzed by scanning electron microscopy (SEM) before (control) and after LNI pretreatment. Both pretreated and non-pretreated samples were metallized by gold evaporation on their surfaces with an argon plasma evaporator (108 Manual Sputter Coater, Cressington Scientific Instruments, Watford, UK). The metallized samples were analyzed with a SEM JEOL microscope (JSM-6360LV, Tokyo, Japan). The analyses were carried out in duplicate.

#### 2.2.3. Drying Experiments

Whole fruit samples were dried by using three drying methods: hot-air-drying (AD), vacuum drying (VD) and freeze-drying (FD). A laboratory hot-air tray dryer (Model UOP8-G, Armfield, Hampshire, UK) was used for AD under constant conditions of 50 °C and 1 m/s air velocity. Air speed and temperature were measured continuously using an anemometer (LCA 6000 Airflow Development Ltd., Andover, NJ, USA) and T-type thermocouples (Omega Engineering Inc., Laval, QC, Canada), respectively. For VD, a lab-scale Isotemp vacuum oven (Model 281A, Fisher Scientific, Etobicoke, ON, Canada) was used to dry berries at 50 °C and 25″ Hg vacuum. Finally, FD was performed in a laboratory freeze-dryer (Freeze-mobile 25 L, Virtis Company, New York, USA) at a constant heating plate temperature of 25 °C, a condenser temperature of −85 °C and at less than 30 mTorr.

Drying curves were obtained by the periodic weighing of berry samples at different processing times of 1, 2, 4, 6, 8, 16, 24 and 48 h with a Sartorius balance (Model BCE2202I-1S, Göttingen, Germany). Dried berry fruits were stored immediately after AD, VD or FD in desiccators in the presence of P_2_O_5_ for further analysis. The moisture content was determined as a function of drying time as described later.

The dry mass of the samples was determined in an Isotemp vacuum oven (Model 281A, Fisher Scientific) in the presence of a desiccant (Drierite^®^) at 60 °C for 48 h. Water content in a dry basis was then determined at different times by the use of Equation (1):

(1) $$X=(m-m_{s})/m_{s}$$

where *X* is the water content in a dry basis (kg water/kg dry matter), and *m* and *m_s_* are the weight of the sample at time t and the dry mass, respectively. Then, drying curves were plotted as moisture content ratio *X/X*_0_ as a function of time, where *X*_0_ is the initial water content (dry basis).

The change in moisture content during drying could be described, for different drying methods, by the simple two-parameter decaying exponential Equation (2) [34]:

(2) $$\frac{X}{X_{0}}=k_{1}\;\exp\;\left(-k_{2}\;t\right)\;$$

where parameters *k*_1_ (--) and the rate constant *k*_2_ (h^−1^) are fitting parameters that can be obtained from adjusting Equation (2) to the experimental data with non-linear regression using Sigmaplot (Version 12.5, Systat Software Inc., San Jose, CA, USA). Increases in drying efficiency using LNI pretreatment could be estimated by comparing the drying rate constant *k*_2_ for different treatments. The residual sum of squares (SSE) for the fitting of Equation (3) to the experimental data was used to assess the adequacy of the regression:

(3) $$SSE=\sum_{i=1}^{n}{\left({\left(X/X_{0}\right)}_{exp}-{\left(X/X_{0}\right)}_{pre}\right)}^{2}$$

where *(X/X*_0_*)_exp_* and *(X/X*_0_*)_pre_* are the experimental and predicted values of the moisture content ratio.

#### 2.2.4. Quality Assessment

Fruits were grinded into a puree to proceed with initial characterization in terms of water content, soluble solids content (°Brix), water activity and pH. For this, water content was gravimetrically obtained as described earlier, a digital refractometer (Reichert AR 200, Reichert Inc., Depew, NY, USA) was used to determine the °Brix of the purees and an Aqualab (Aqualab, Meyer Service & Supply Ltd., ON, Ontario Canada), beforehand calibrated with saturated salt solutions of known relative humidities, for the determination of water activity. Finally, the pH of the different pureed berries was obtained with a pH meter, Symphony SP20 (VWR Symphony, Thermo Orion, West Chester, PA, USA), calibrated with buffer solutions with pH 4.0 and pH 7.0.

A visual inspection of the berries was conducted before and after LNI pretreatment and after the drying processes. Pictures of the initial samples and the samples after LNI and drying (all samples for each berry are in the same picture for comparison purposes), were taken with a digital camera. Digital pictures were analyzed using an automatic image processing method with ImageJ 2.0 software (Creative Commons license). The images were calibrated by the application “Set scale” of the same software. Subsequently, the volume reduction was estimated with the application “Analyze particles” of the software. Finally, color changes in the fruits were analyzed with the application “Color picker” of the software to establish a representative color analysis, the color of each fruit appearing in the image was determined at ten different points [35]. The results are presented in terms of CIELAB color parameters L (lightness), a* (red/green value), b* (blue/yellow value), ∆L (lightness difference) and ∆E (total color difference).

#### 2.2.5. Statistical Analysis

The data were expressed as the mean ± SD of triplicate experiments (*n* = 3). Statistical analysis was carried using one-way analysis of variance (ANOVA) with Minitab 16.0 software (Minitab Inc., State College, PA, USA). The significant difference between means was evaluated using the Tukey test for a means comparison. A *p*-value of less than 0.05 was considered statistically significant.

## 3. Results and Discussion

Table 1 presents the initial physicochemical characteristics of the three berries under study. As expected, seabuckthorn fruits presented the smallest diameter followed by blueberries, and finally, green grapes. In terms of epicarp thickness, blueberry showed the thickest values, almost double than for seabuckthorn fruits which are the smallest. The epicarp thickness values in Table 1 are in a similar range of the literature data found for apple, grape, guava and tomato [36,37,38]. It should be pointed out that the thickness values presented in Table 1 are for the fruit epicarp, commonly called ‘skin’, which is significantly thicker than the proper cuticle (Figure 1). By way of illustration, the cuticle thickness of Highbush blueberries was found to be approximately 0.05 mm [11], which is 10 times smaller than the blueberry epicarp found in this study (Table 1).

As shown in Table 1, the average moisture content of seabuckthorn berries was 84.03%, which is closer to the moisture level of blueberries and slightly higher than that of green grapes. These values agree with previously published data for fresh seabuckthorn [39,40,41], blueberries [11,42] and grapes [43,44]. Green grapes presented the highest soluble solid level (20.03 °Brix) followed by Highbush blueberries and seabuckthorn fruits. For green grapes, °Brix is usually used as a fruit ripeness index to determine the right harvest time to obtain a good wine fermentation (°Brix > 24.3) [44,45]. The °Brix value of seabuckthorn berries was similar to the average of 11.4°Brix reported in the literature for Indian Summer cultivar [39,41] while the °Brix for blueberries remained in the same range than values reported for other similar varieties [42]. Regarding the pH values, the lowest pH was observed in seabuckthorn berries, followed by highbush blueberries and green grapes. Similar pH data were published in the literature [11,41,44].

**Table 1:** Initial physicochemical characterization of berries. Different lowercase letters are significantly different (*p* < 0.05).

	Size (Main Diameter, mm)	Epicarp Thickness (mm)	Wax Content Range (mg/cm^2^)	Water Content (kg Water/kg Total Mass)	°Brix	pH	a_w_
Seabuckthorn	8.8 ± 0.9 a	0.362 ± 0.029 a	---	84.03 ± 0.38 a	10.03 ± 0.38 a	2.81 ± 0.01 a	0.99 ± 0.01 a
Blueberry	13.2 ± 1.8 b	0.627 ± 0.040 b	30–200 a [11,46,47]	83.57 ± 2.16 a	12.27 ± 0.06 b	3.31 ± 0.01 b	0.99 ± 0.01 a
Green grape	23.5 ± 2.2 c	0.442 ± 0.063 a	15–50 a [48,49,50]	77.17 ± 2.29 b	20.03 ± 0.07 c	3.47 ± 0.01 c	0.98 ± 0.01 a

**Figure 3:**
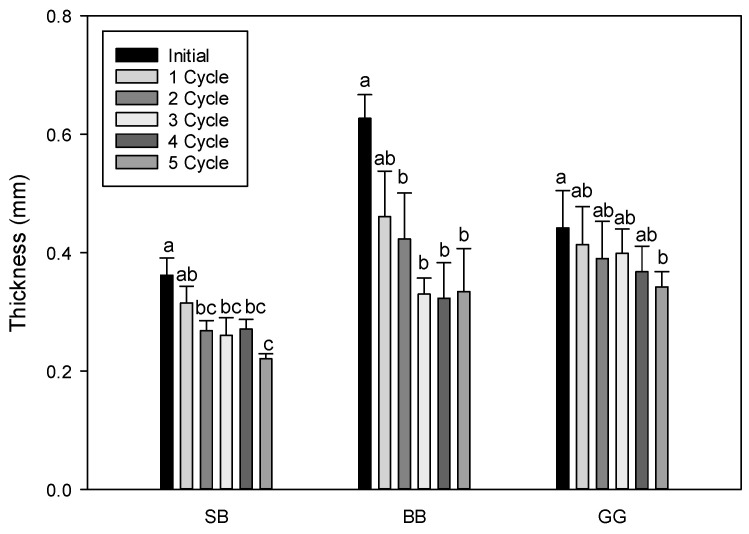
Skin thickness as a function of number of liquid N_2_ cycles (SB = seabuckthorn, BB = blueberries, GG = green grapes). Note: Values are mean ± SD. Means in the same group (fruit sample) with different lowercase superscripts are significantly different (*p* < 0.05).

Figure 3 shows the epicarp thickness reduction as a function of LNI treatment cycles, compared to the initial thickness. Significant differences were observed after two cycles for seabuckthorn and blueberry, and after five cycles for green grape, indicating a significant decrease in epicarp thickness for all berry types upon immersion in liquid nitrogen. Increasing the cycle numbers showed a positive effect on thickness reduction. As an example, seabuckthorn revealed a maximum decrease of 39% after five LNI cycles, while blueberry and green grape revealed a 47% and 23% decrease, respectively.

Differences in the final thickness reduction for different berries could be related to variations in their epicarp composition, the amount of intracuticular versus epicuticular waxes, and specific total wax quantity for each berry. Table 1 indicates the literature data for the total wax amount for blueberry and grape (no data could be found for seabuckthorn). Although a rough estimation from different literature sources, blueberries seem to have noticeably higher amounts of total wax than grapes, which correlates well with the higher reduction in thickness found after LNI.

**Figure 4:**
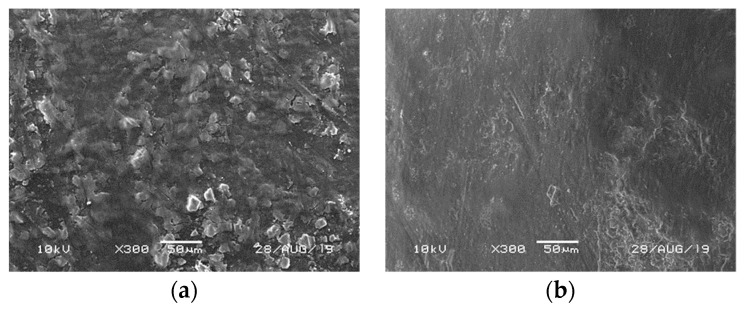
SEM micrographs of blueberry surface before (**a**) and after (**b**) the cryogenic pretreatments (five thermal shocks in liquid nitrogen).

Figure 4a shows an example for blueberries of a SEM image of the waxy epicuticular layer covering their surface. It is a dense network of waxes with amorphous structure. After LNI, this network of waxes disappeared as depicted in Figure 4b, with smooth surfaces indicating cuticular dewaxing. It should be noted that a similar behavior was observed in the seabuckthorn and green grape samples, although specific images for these samples are not provided. The scalping of cuticular wax by immersions in liquid nitrogen might be attributed to the mechanical action of nitrogen bubbles forming at the contact site between the warmer blueberry surface and the liquid nitrogen [11]. This may have partially contributed to a decrease in epicarp thickness as observed earlier. Pham et al. [51] concluded that liquid nitrogen could extract most superficial waxes (epicuticular) from different grains, while n-hexane showed penetration into the cuticle, extracting both epicuticular and intracuticular waxes.

**Figure 5:**
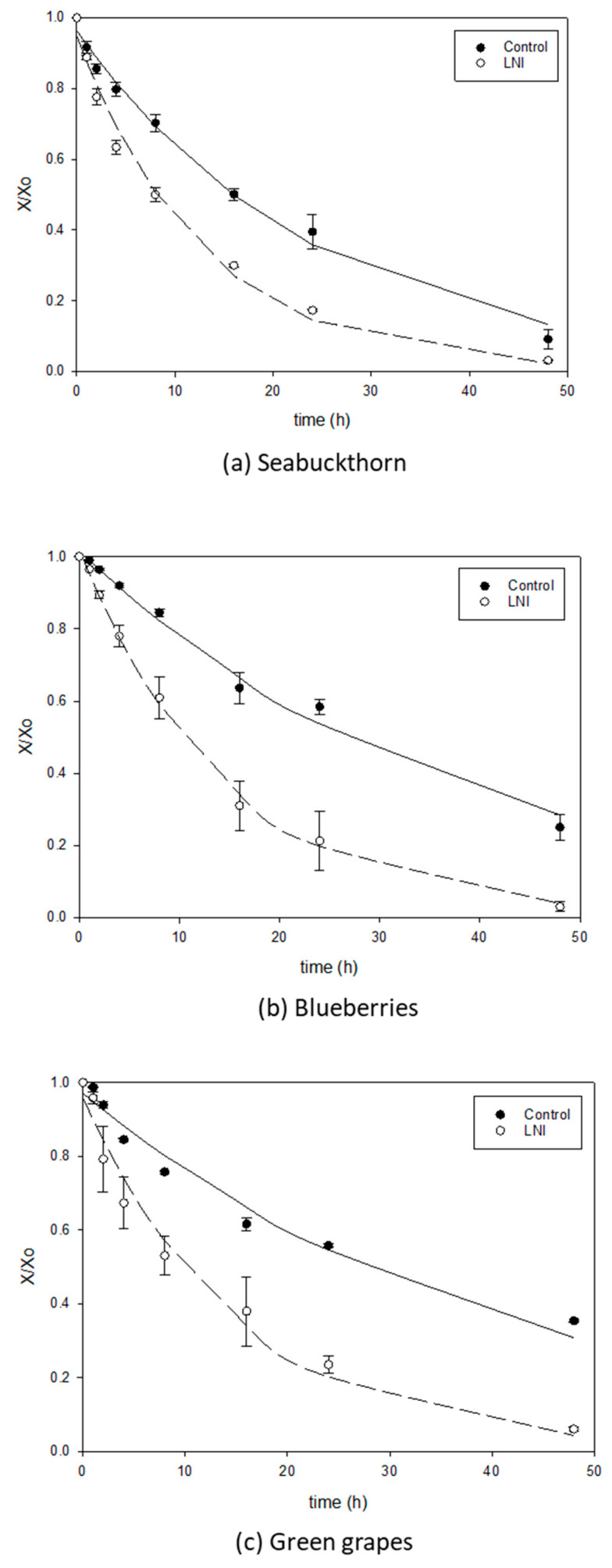
Air-drying curves for seabuckthorn (**a**), blueberries (**b**) and green grapes (**c**), untreated (control) versus pretreated (five cycles of LNI pretreatment) samples.

**Figure 6:**
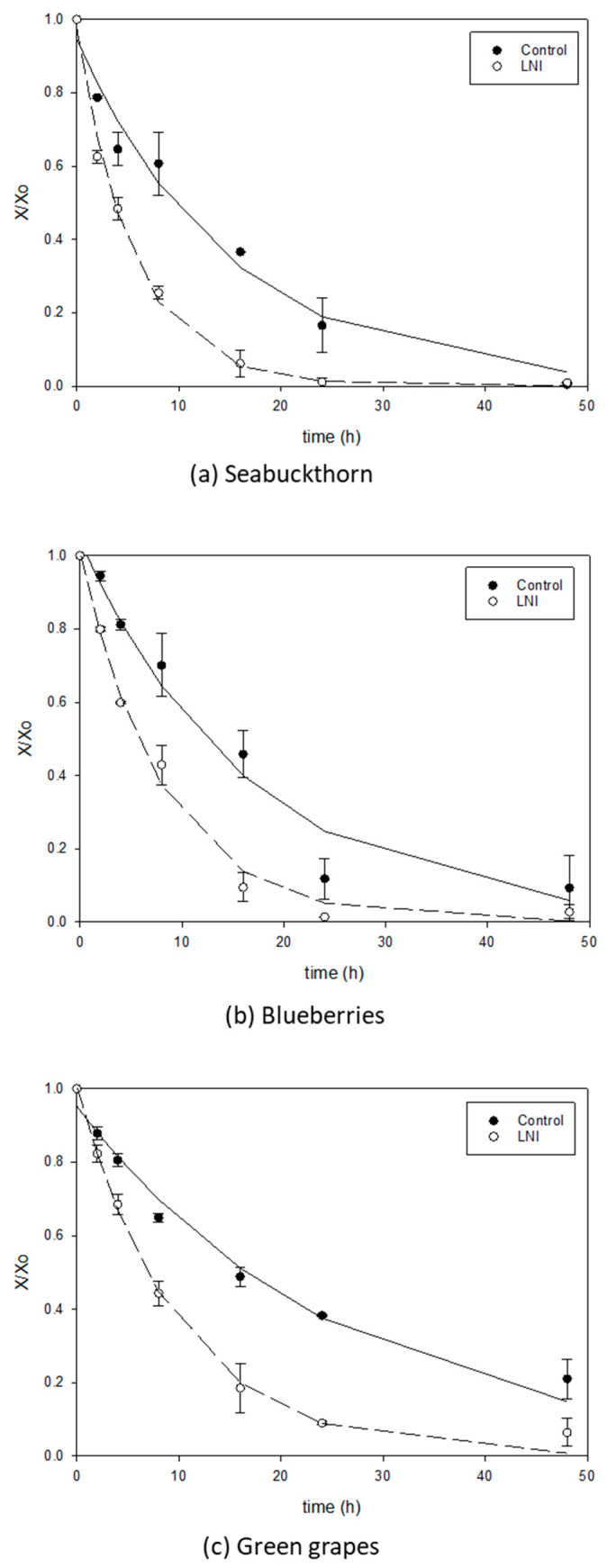
Vacuum-drying curves for seabuckthorn (**a**), blueberries (**b**) and green grapes (**c**), untreated (control) versus pretreated (five cycles of LNI pretreatment) samples.

**Figure 7:**
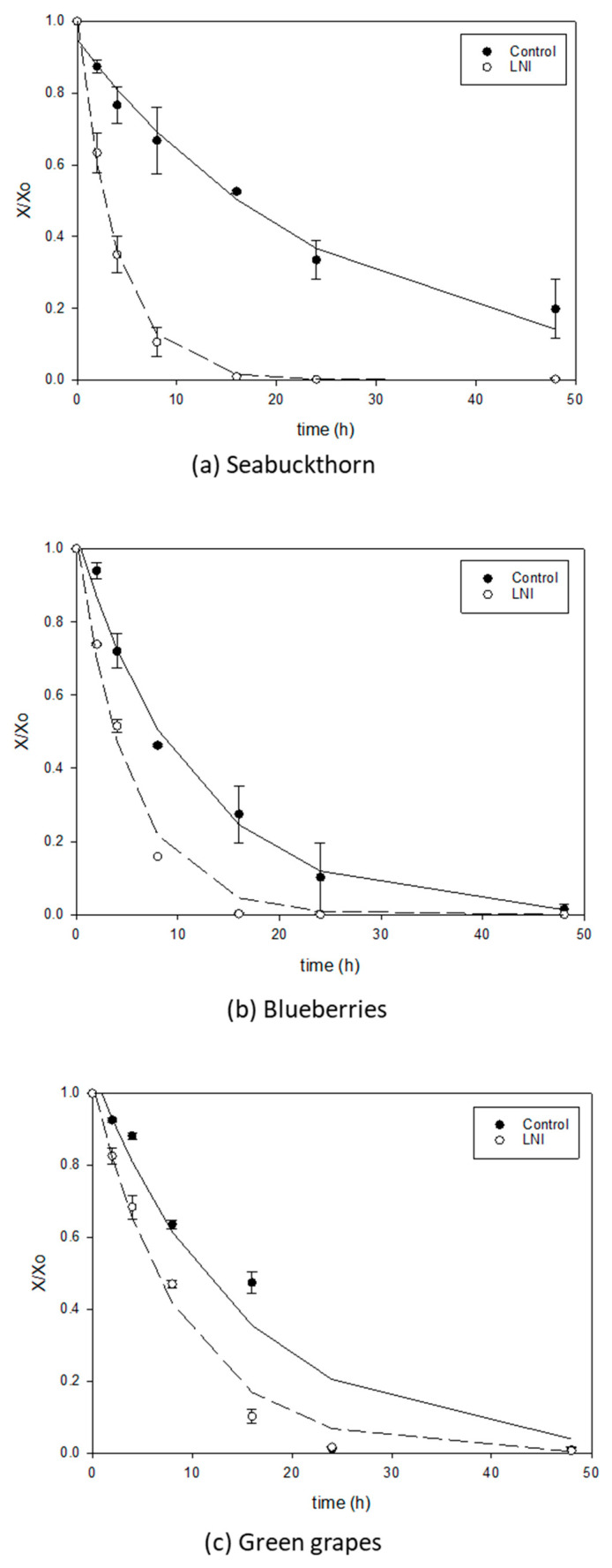
Freeze-drying curves for seabuckthorn (**a**), blueberries (**b**) and green grapes (**c**), untreated (control) versus pretreated (five cycles of LNI pretreatment) samples.

Figure 5, Figure 6 and Figure 7 show the comparison of drying curves (with or without five cycles of LNI pretreatment) for air-drying, vacuum-drying and freeze-drying, respectively. All the drying curves present the typical decaying exponential tendency with a steep decrease in the first hours of drying, followed by a slow down when bound water is predominant and more difficult to separate from the matrix. Air-drying seems to be the slowest drying technology for berries without treatment, as can be seen in Figure 5 (filled symbols) where most curves do not reach equilibrium even after 50 h of drying. These lengthy drying times agree with the literature data for the air-drying of whole berries. As an example, the air-drying time (1 m/s and 50 °C) for green grapes was reported to be 82 h [52], even after chemically pretreating the skin. For blueberries, 22.5 h were reported for air-drying at 50 °C and 1.3 m/s [53] (air speed higher than in the present study). Vacuum application has a positive impact on improving drying rates for untreated berries, as can be observed in Figure 6 and Figure 7 (filled symbols) for vacuum-drying and freeze-drying, respectively. The vacuum-drying times for seabuckthorn and blueberries (no pretreatment) were found to be in the order to 40 h (Figure 6a,b, respectively), while for green grapes, it was 50 h (Figure 6c). The literature data for the vacuum-drying of untreated blueberries indicated 18 h of drying time, a lower value than the one obtained in the present study [53]. However, although the drying temperature was the same in both studies, the vacuum applied in the work carried out by Akcicek et al. [53] was 10-fold lower than the one in this study (2 versus 25″ Hg, respectively), which might be the reason for the lower drying times.

Liquid nitrogen pretreatment had a significant effect on improving drying rates for all berries and drying methods under study, which can be observed in Figure 5, Figure 6 and Figure 7, with empty versus filled symbols corresponding to pretreated and untreated berry samples, respectively. Ketata et al. [11] observed a reduction in osmotic dehydration time from 45% to 65%, depending on blueberry types (commercial versus wild blueberries), for liquid nitrogen immersion-treated samples when compared to the control (untreated) blueberries. The dewaxing of the blueberry skin due to liquid nitrogen immersions was pointed out as responsible for the great acceleration of the process. For chemically pretreated berries, Doymaz and Pala [54] reported notable reductions in air-drying times for grapes at 60 °C air temperature when berries were previously dipped in potassium carbonate solution (drying time 1320 min) or alkaline emulsion of ethyl oleate (1230 min) compared to the untreated grapes (2880 min).

Table 2 summarizes the results of the fitting of Equation (2) to the experimental data on drying curves for treated and untreated berries prior to AD, VD and FD, together with the residual sum of squares presented as a percentage (SEE (%), calculated from Equation (3)). In Figure 5, Figure 6 and Figure 7, simulations of the correlation (Equation (2)) with corresponding fitted parameters (Table 2) are displayed together with the experimental data. As can be seen from the excellent agreement between the simulations and experimental data (Figure 5, Figure 6 and Figure 7), and from the goodness of fitting observed from the lower SSE percentages shown in Table 2 (for most cases, lower than 5%), a double parameter decaying exponential represents remarkably well most of the drying data for berries with or without pretreatment. The higher SSE values found for the untreated blueberries (vacuum-drying, Figure 6b) and untreated green grapes (freeze-drying, Figure 7c) could be due to the combination of the high impermeability of the skin and vacuum application. Water pressure inside the samples increases due to the impossibility of water to leave the matrix, and thus the skin bursts, releasing water instantaneously, as shown by a rapid decrease in water content at 24 h for vacuum-drying and freeze-drying in Figure 6b and Figure 7c, respectively. As expected, parameter k_1_ in Table 2 was close to 1 [34], while rate parameter k_2_ can help further to assess the drying process. The lowest k2 values were found for hot-air-drying of untreated (control) blueberries and green grapes (less than 0.03 h^−1^, Table 2), confirming the poor performance of this type of drying process for berries. LNI pretreatment has an impressive positive effect on accelerating drying rates, as can be seen from Table 2 from the percentage increase (%A) for parameter k_2_, ranging from 60 to 547% for all drying processes under study. The highest increase in parameter k_2_ due to LNI pretreatment was found for the freeze-drying of seabuckthorn berries (from 0.04 to 0.259 h^−1^) for which the drying time was reduced to just 16 h (Figure 7a).

The final relative moisture content (after 48 h drying) is shown as well in Table 2, where the samples that are considered ‘dry’ are denoted with an asterisk. As can be observed, most LNI-treated berries were dried after 48 h. LNI-treated green grapes were only dried by the freeze-drying process, which could be due to the bigger size of this berry compared to the other berries (Table 1) and the longer drying times necessary to dry grapes for vacuum- and particularly air-drying.

Table 3 shows the quality assessment (visual, shrinkage reduction and color changes) for air-dried, vacuum-dried and freeze-dried berries, with and without LNI pretreatment. As expected, shrinkage reduction was the lowest for all freeze-dried berries (LNI-pretreated or not), while in general, air-dried samples shrunk the most, up to 62% for LNI-pretreated blueberries (Table 3). Berries with a delicate structure and high levels of water content are very difficult to dehydrate by classical methods [32,55], especially during air-drying when a collapse causes considerable damage to their physical structure [56]. Berries being very susceptible to skin rupture during air-drying may cause severe fruit bleeding during drying. The freeze-drying process, however, is known for producing low-shrinkage berry products [57].

Color is one of the key quality evaluation indices for berries. The VD- and AD-treated samples showed lower L*, a* and b* values compared to the initial samples, especially green grapes samples. The decrease in the L* value may also be related to the enzymatic and non-enzymatic browning of berries during drying [58]. Berries have a high content of saccharides, and drying may release them from the cell structure and fragment them into small molecules, thereby promoting the non-enzymatic browning of berries. Regarding color change, the lowest ΔE was still attained by freeze-dried berries. This was due to the vacuum environment and low temperatures inhibiting enzymatic browning and the occurrence of the Maillard reaction. For the vacuum- and air-dried berries, color changes were similar and higher than for the freeze-dried berries, attributed to the reduction in brightness due to the Maillard reaction owing to higher temperatures, longer drying times and oxidation. Similar color results were found for seabuckthorn berries by [59].

**Figure 8:**
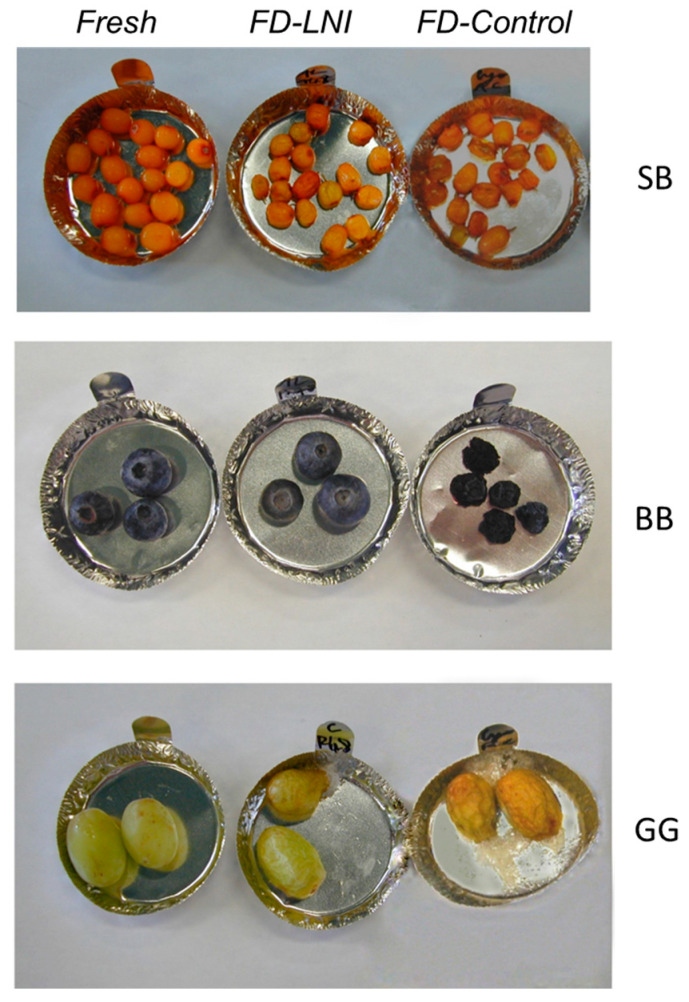
Photography of fresh and freeze-dried berries with/without (control) LNI pretreatment.

In general terms, freeze-drying provides the best quality dried berries. The visual inspection of the berries correlates well with quantitative shrinkage and color determinations, as shown in Figure 8 for the freeze-drying process. From this figure and Table 3, blueberries pretreated with liquid nitrogen immersion presented the highest quality from all the berries after freeze-drying.

It should be considered though that freezing food materials prior to drying is an integral part of the freeze-drying process, but it could cause additional quality losses if the frozen material is then transformed through another drying method such as vacuum- or air-drying. Thawing could increase shrinkage and color deterioration during the vacuum- or air-drying of frozen berries.

## 4. Conclusions

Liquid nitrogen pretreatment reduced epicarp thickness for all berries, with maximal thickness reduction (47%) for blueberries after five cycles of LNI, mainly due to the dewaxing of the berry surface observed by SEM. The freeze-drying times for these berries were shorter than for other drying methods under study. Drying constant k_2_ increased markedly with LNI pretreatment for all drying methods, but especially for FD, for which it increased 1.5 to 6.5 times for different fruits. This has an interesting economic impact as FD is well known for being an expensive drying process due to the high energy required to maintain vacuum. In terms of quality after drying for LNI-treated fruits, FD berries presented the lowest color change and minimal shrinkage. On the other hand, the control samples exhibited a better or similar quality after processing for VD and AD compared to the LNI-treated fruits. The quality of the dried product after LNI treatment also depended on the type of berries. In this case, the most beneficial effect is definitely achieved with BB-FD due to the low shrinkage (2.9%) and the low ∆E value (3). Liquid nitrogen immersion proved to be an effective pretreatment in accelerating drying rates of whole berries with premium quality, and by leaving no subsequent residues, it represents an interesting alternative to traditional pretreatments.

## Figures and Tables

**Table 2:** Drying curves kinetic parameters (SB = seabuckthorn, BB = blueberries, GG = green grapes).

		(*X/X*_0_)_48 h_	*k*_1_	*k*_2_ (h^−1^)	*%A* ^1^	*SEE* (%)
		AD				
SB	Control	0.091	0.964	0.041	90.24	3.2
	LNI	0.031 *	0.948	0.078		3.9
BB	Control	0.25	1.018	0.027	151.85	2.9
	LNI	0.030 *	1.02	0.068		1.9
GG	Control	0.353	0.971	0.024	170.83	4.1
	LNI	0.06	0.959	0.065		5.3
		VD				
SB	Control	0.004 *	0.946	0.067	170.15	5.8
	LNI	0.009 *	0.975	0.181		2.9
BB	Control	0.093	1.041	0.06	106.67	7.3
	LNI	0.028 *	1.011	0.124		3.9
GG	Control	0.21	0.952	0.039	158.97	4.3
	LNI	0.064	1.006	0.101		2.7
		FD				
SB	Control	0.198	0.95	0.04	547.50	4.4
	LNI	0.003 *	1.012	0.259		1.8
BB	Control	0.015 *	1.04	0.09	116.67	4.4
	LNI	0.001 *	1.042	0.195		4.4
GG	Control	0.009	1.066	0.069	63.77	11.2
	LNI	0.007 *	1.03	0.113		4.8

* indicates samples that are dry after 48 h; ^1^ %A indicates percentage of acceleration due to LNI pretreatment, %A = ((k_2LNI_ − k_2Control_)/k_2Control_) × 100.

**Table 3:** Quality characterization of dried berries.

		Visual Aspect	Area (cm^2^)	Shrinkage (%)	L	a*	b*	∆L	∆E
		Control (no pretreatment)							
SB	Initial	5/5	0.81 ± 0.12 a	---	66 ± 2 a	39 ± 6 a	69 ± 2 a	---	---
	FD	2/5 Color Change, Shrinkage	0.63 ± 0.16 ab	22 ± 3 a	58 ± 5 b	37 ± 5 ab	60 ± 5 b	−8 ± 3 a	18 ± 5 a
	VD	2/5	0.47 ± 0.16 bc	42 ± 7 b	48 ± 6 c	34 ± 3 b	48 ± 7 c	−18 ± 6 b	30 ± 8 b
	AD	1/5 Color Change, Shrinkage	0.35 ± 0.09 c	56 ± 8 c	35 ± 5 d	21 ± 3 c	31 ± 3 d	−33 ± 7 c	55 ± 10 c
BB	Initial	5/5	1.87 ± 0.17 a	---	39 ± 3 a	−0.71 ± 0.02 a	0.72 ± 0.14 a	---	---
	FD	2/5 Shrinkage	0.97 ± 0.02 c	16 ± 3 b	35 ± 3 ab	−0.4 ± 0.05 b	−2.4 ± 1 c	−8 ± 1 ab	8.2 ± 0.5 a
	VD	1/5 Shrinkage	1.6 ± 0.3 ab	48 ± 5 a	28 ± 2 c	0.07 ± 0.05 c	−0.20 ± 0.03 b	−11 ± 2 a	11 ± 1 b
	AD	1/5 Shrinkage	1.1 ± 0.5 bc	42 ± 8 a	32 ± 1 b	0.21 ± 0.05 d	−2.9 ± 0.9 c	−6.9 ± 0.3 b	7.9 ± 0.5 a
GG	Initial	5/5	5.1 ± 1.1 a	---	60 ± 1 a	−5.4 ± 1.9 a	49 ± 3 b	---	---
	FD	1/5 Browning, Shrinkage	4.2 ± 0.6 a	19 ± 5 a	60 ± 3 a	9 ± 2 b	55 ± 1 a	−0.56 ± 0.12 a	15 ± 2 a
	VD	0/5 Browning, Shrinkage	3.7 ± 0.9 a	28 ± 4 a	27 ± 2 b	10 ± 2 b	15 ± 5 c	−33 ± 2 b	50 ± 4 b
	AD	0/5 Browning, Shrinkage	3.6 ± 0.9 a	28 ± 4 a	27 ± 1 b	7.0 ± 0.2 c	10.2 ± 0.9 c	−33 ± 1 b	53 ± 1 b
		Liquid nitrogen immersion pretreatment							
SB	Initial	5/5	0.97 ± 0.17 a	---	60 ± 6 a	38 ± 4 a	54 ± 6 a*	---	---
	FD	4/5	0.7 ± 0.2 ab	32 ± 5 a*	64 ± 4 a	32 ± 3 a	58 ± 4 a	4 ± 1 a*	15 ± 3 a*
	VD	0/5 Color Change, Collapse	0.55 ± 0.16 b	44 ± 6 a	36 ± 3 b	16 ± 3 b*	20 ± 5 b*	−23 ± 3 b	47 ± 7 b*
	AD	0/5 Color Change, Collapse	0.50 ± 0.11 b*	48 ± 6 a	32 ± 3 b	9 ± 3 b*	12 ± 3 b*	−28 ± 2 b	57 ± 6 b
BB	Initial	5/5	2.34 ± 0.17 a*	---	26 ± 3 a*	−0.21±0.02 b*	0.15 ± 0.03c*	---	---
	FD	5/5	2.3 ± 0.4 a*	2.9 ± 0.2 a*	26 ± 3 a*	−0.50 ± 0.08 a	0.96 ± 0.12 a*	−0.77 ± 0.05 c*	3 ± 1 c*
	VD	0/5 Leakage	1.5 ± 0.2 b	35 ± 6 b*	20 ± 1 b*	0 d*	0 d*	−6 ± 1 a*	6 ± 1 ab*
	AD	3/5 Good Color, Shrinkage Wrinkles	0.9 ± 0.1 c	62 ± 15 c*	25 ± 2 a*	−0.17 ± 0.02 c*	0.22 ± 0.04 b*	−1.6 ± 0.5 b*	4 ± 1 bc*
GG	Initial	5/5	5.8 ± 0.3 a	---	59 ± 1 a	−8 ± 2 a	47 ± 4 a	---	---
	FD	3/5 Good Color, Some Shrinkage and Wrinkles	4.4 ± 0.4 b	24 ± 3 a	54 ± 2 b*	−9 ± 1 a*	45 ± 3 a*	−5 ± 1 b*	6 ± 1 b*
	VD	0/5 Browning, Shrinkage	5.3 ± 0.3 a*	7 ± 1 b*	27 ± 1 c	3.4 ± 0.3 c*	0.6 ± 0.1 b*	−33 ± 1 a	58 ± 1 a*
	AD	0/5 Browning, Shrinkage	4.2 ± 1.1 ab	27 ± 3 a	24 ± 1 d	0.10 ± 0.03 b*	1.1 ± 0.2 c*	−35 ± 1 a	58 ± 1 a*

Different letters indicate significant differences between the different drying treatments, while the asterisk (*) indicates significant differences between samples with and without pretreatment.

## Data Availability

The original contributions presented in the study are included in the article, further inquiries can be directed to the corresponding author.
